# Is histone H2B deubiquitylation via ATXN7L3-dependent enzymes a new mechanism disrupted in neurodevelopmental disorders?

**DOI:** 10.1007/s10048-026-00954-8

**Published:** 2026-11-07

**Authors:** Abhinav Anand, Francisco Bustos

**Affiliations:** 1 Division of Biomedical & Translational Sciences, Sanford School of Medicine, University of South Dakota, Vermillion, SD 57069 USA; 2 Center for Genetics and Rare Diseases, Sanford Research, Sioux Falls, SD 57104 USA; 3 Department of Pediatrics, Sanford School of Medicine, University of South Dakota, Sioux Falls, SD 57105 USA

**Keywords:** Histone H2B, Ubiquitin, Deubiquitylases, ATXN7L3, USP27X, Neurodevelopmental Disorders

## Abstract

Neurodevelopmental disorders are lifelong debilitating conditions that affect cognition and originate during embryonic development. Being heterogeneous and with a variety of etiologies, most of these disorders have no targeted treatment or cures, due to our lack of knowledge on the underlying molecular mechanisms disrupted in their pathogenesis. An emerging epigenetic mechanism recently associated with neurodevelopmental disorders is ubiquitylation of histone H2B. This posttranslational modification controls gene expression during neurodevelopment, where histone H2B ubiquitylation levels are tightly regulated by multiple deubiquitylase protein complexes ensuring correct gene expression. Components of deubiquitylase complexes regulating histone H2B ubiquitylation are the protein scaffolds ATXN7L3 and ENY2, and the deubiquitylating enzymes USP22, USP27X, and USP51. Recent clinical studies have highlighted a potential role of H2B ubiquitylation in human neurodevelopment, including the identification of neurodevelopmental disorders associated with gene variants in the *ATXN7L3*, *USP27X* and *USP51* genes. Here, we summarize our knowledge of the functions of deubiquitylase complexes that regulate histone H2B ubiquitylation and their components in neurodevelopment, and describe *ATXN7L3*, *USP27X*, and *USP51*-associated disorders, proposing future directions on the required research on their fundamental biology that could lead to targeted therapies.

## Introduction

Neurodevelopmental disorders are a heterogeneous group of neurological conditions [1, 2]. On average, 2–3% of the population is affected by neurodevelopmental disorders [3, 4]. With no cure and current treatments prioritizing symptom mitigation, patients who are affected by this debilitating disease experience diminished cognition and adaptive function that begins from infancy and lasts for a lifetime [5]. These predicaments motivate ongoing investigation into the molecular mechanisms behind neurodevelopmental disorders to potentially shed light on novel therapeutic targets.

The ubiquitin system plays key roles in protein biology. This system regulates proteostasis, localization, and interaction regulating crucial cellular processes such as transcription, autophagy, cell cycle, and differentiation [6]. Ubiquitylation refers to the post-translational modification (PTM) of protein substrates via a three-step enzymatic process; leading to the addition of a ubiquitin motif – a 76-amino acid protein added to lysine residues within target proteins [7, 8]. The three steps of ubiquitin addition are activation, conjugation, and ligation, mediated by E1, E2, and E3 enzyme families respectively [9]. Just as ubiquitin can be added, so can it be removed. This action is mediated by the deubiquitylase (DUB) class of enzymes, the largest family of which are the ubiquitin-specific proteases (USPs) [10]. Prior literature demonstrates that neurodevelopmental disorders are frequently associated with genetic perturbations to DUBs [11]. Symptoms vary widely depending on the downstream protein substrates but can range from developmental delay and intellectual disability to craniofacial features, seizures, hypotonia, and congenital abnormalities [12–15]. From a biochemical perspective, however, little is known about the effect of these DUB variants on structural form and biological function.

A critical and highly conserved ubiquitylation process modifies histone H2B (H2Bub1) at lysine 120 (K120) with a single ubiquitin moiety in a dynamic process that helps to govern transcription initiation [16–21]. The E3 ligases RNF20 and RNF40 catalyze histone H2B ubiquitylation [22–27]. Addition of ubiquitin to H2B generally promotes gene expression through chromatin remodeling via Histone H3 lysine 4 trimethylation (H3K4me3) and histone H3 lysine 79 dimethylation (H3K79me2) by COMPASS (Complex of Proteins Associated with Set1) and DOT1L respectively in gene bodies, thereby promoting transcription and gene elongation courtesy of stimulated RNA polymerase II processivity via FACT (facilitates chromatin transcription) complex recruitment. The FACT complex then causes conformational changes in nucleosome structure to permit RNA polymerase II to bypass the chromatin barrier [28–31]. However, H2B ubiquitylation can also repress gene expression at quiescent promoters via stabilization of the nucleosome and subsequent inhibition of transcript initiation complex formation [31].

**Fig. 1:**
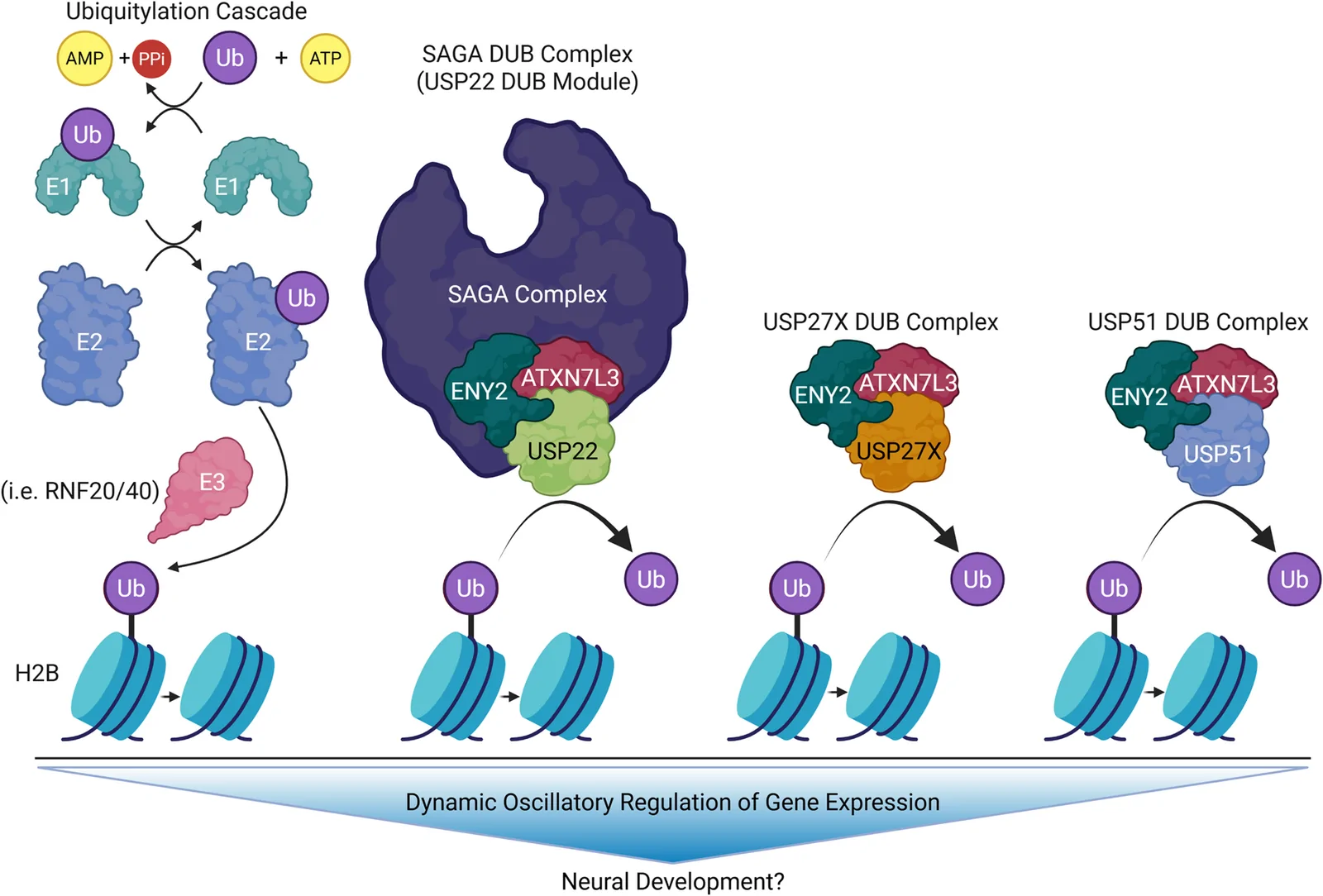
Histone H2B ubiquitylation levels regulated by ATXN7L3-dependent DUB complexes may shape human neural development. Accurate gene expression depends on a rapid and dynamic balance between ubiquitin addition and removal. The E1 through E3 cascade of enzymes add monoubiquitin motifs to the tail of histone H2B for regulation of gene expression, while ATXN7L3-dependent DUB complexes that contain USP22 (SAGA DUB module), USP27X or USP51 which have histone H2B substrate specificity remove these same ubiquitin motifs. Association of components of these complexes with genetic neurodevelopmental disorders suggests that this dynamic back-and-forth allows for rapid heterochromatin and euchromatin states that permit the accurate and oscillatory expression of critical genes that could shape human neurodevelopment. Created in BioRender. A, A. (2026) https://BioRender.com/ot9dkd8 [76]

Conversely, The Spt-Ada-Gcn5 acetyltransferase (SAGA) is a transcriptional activator complex that catalyzes H2Bub1 deubiquitylation via its DUB module consisting of ataxin 7 like 3 (ATXN7L3), USP22, and enhancer of yellow 2 transcription factor homolog (ENY2). Another protein, ataxin 7 (ATXN7), connects the DUB module to the overall SAGA complex. The proper SAGA subunit assembly and organization are critical for its efficacious DUB activity [18, 21, 32–40]. Besides USP22, USP27X and USP51 constitute alternative ATXN7L3-dependent DUB complexes that control H2Bub1 deubiquitylation and gene expression [41] (Fig. 1). These complexes broadly regulate H2Bub1 across the genome, mediating the majority of H2Bub1 turnover at actively transcribing genes. Consistently, their disruption drives genome-wide increase in H2Bub1 levels [41]. Although DUB complexes containing USP3, USP7, USP12, or USP46 can also catalyze H2Bub1 deubiquitylation, they regulate more restricted, context dependent transcriptional programs [42–46]. Therefore, active transcription depends on tightly regulated H2Bub1 turnover rather than a continuous ubiquitylated state.

Recent research highlights familial *USP27X* and *USP51* gene variants as well as *de novo ATXN7L3* gene variants associated with neurodevelopmental disorders, and new research describes variants via their clinical and biochemical characteristics of disease manifestation. X-linked intellectual disorder 105 (XLID 105), is associated with variants in the *USP27X* gene and presents as a syndrome with a wide range of symptoms – from intellectual disability to speech delay, autistic behaviors, dysmorphic features, and microcephaly [12, 47]. On the other hand, clinical manifestations of Harel-Tora neurodevelopmental syndrome, associated with *ATXN7L3* variants, also vary widely depending on variant. Some symptoms include, but are not limited to: Abnormal head size, global developmental delay, congenital cardiac defects, and dysmorphic features [48]. However, due to the relative recency in investigating *USP27X*, *USP51* and *ATXN7L3* variants’ pathogenesis, it is still not clear whether and how they are causative for some or all these symptoms.

Due to the nature of ATXN7L3-dependent DUB complexes as a mechanistically related network that accounts for most H2Bub1 regulation across the genome, this review intends to disclose what is currently known about their components such as ATXN7L3, USP22, USP27X, and USP51, their role in neurodevelopment, descriptions of noteworthy *USP27X*, *USP51* and *ATXN7L3* associated disorders, and hypotheses on how these complexes may be a potential therapeutic target for those afflicted with gene variants of their subcomponents. In doing so, further investigation into the role of ATXN7L3-dependent DUBs on developmental regulation via H2B deubiquitination may contribute to a better understanding of their antithetic E3 ligase counterparts’ role as well and reinforce the hypothesis that comprehensive developmental homeostasis is achieved through a dynamic and oscillatory relationship between the two components of the ubiquitin cascade.

## The discovery and characterization of USP27X and USP51 as ATXN7L3-dependent DUB complex components

As mentioned above, it was well established that the SAGA DUB module was comprised of ATXN7L3, ENY2 and USP22 forming a complex that catalyzed H2Bub1 deubiquitylation [38]. The idea that additional DUB complexes could deubiquitylate H2Bub1 emerged from seminal experiments by Atanassov and colleagues. They unexpectedly found that loss of USP22 was dispensable for H2Bub1 deubiquitylation although ATXN7L3 was fully required. This suggested that USP22 was not the only interactor partner for ATXN7L3 and ENY2. The existence of additional DUB complexes using alternative DUBs (USP27X and USP51) was discovered through mass spectrometry of purified ATXN7L3, confirming the presence of the non-SAGA components USP27X and USP51. Additionally, overexpression of each of the DUBs resulted in significant reduction of H2Bub1 levels [41]. USP27X and USP51 share significant homology to USP22, with USP27X sharing 82% and USP51 sharing 70%. In addition to the presence of a catalytic domain homologous to USP22 and DUB activity towards H2Bub1, both USP27X and USP51 possess a homologous N-terminal Znf-UBP domain [41], for which USP22 [41] has been demonstrated to be important for interaction with ATXN7L3 and ENY2 [39]. USP27X is unique in that it begins translation from a non-canonical CTG start codon. Evidence for this came from immunoblots in a variety of cell types illustrating a 72 kDa protein as opposed to the documented 50 kDa on databases such as NCBI GenBank [41, 49]. Additional experiments with a mutated CTG codon yielded no endogenous USP27X presence altogether [41].

Using in vitro and in cell experiments, Atanassov and colleagues demonstrated that USP27X and USP51 deubiquitylate H2Bub1 and require ATXN7L3-ENY2 to achieve complete catalytic activity, suggesting that this complex allosterically regulates DUB activity. While USP27X and USP51 do not complex with the SAGA as USP22 does, they do form smaller complexes with ATXN7L3 and ENY2 (Fig. 1). Importantly, USP27X and USP51 compete with USP22 for binding with ATXN7L3 and ENY2, which regulates transcription of particular sets of genes and biological processes related to cell proliferation and tumor growth [41]. Gene specificity could be the result of altered epigenetic marks and subsequent chromatin remodeling juxtapose to target genes. However, this remains speculation, as the exact molecular mechanisms governing protein complex specificity and subsequent histone substrate specificity are currently unknown.

## Roles of ATXN7L3-dependent DUB complex components on neurodevelopment

Many developmental processes are controlled by correct histone H2Bub1 levels [16, 17]. It has been shown that SAGA plays a role in neurodevelopment [33]. Additionally, a study by Liang et al. demonstrated that RNF20 loss-of-function results in impaired astrocytic differentiation from neuronal precursors, accompanied by a reduction in H2Bub1 levels. Further experiments highlighted the regulatory intermediary in this relationship was the complexing of RNF20 with the acetyltransferase MOF to activate Stat3 transcription [50]. Furthermore, Schwann cell-specific RNF40 deletion in mice resulted in dysfunctional myelination driving a neuropathy phenotype with axonal degeneration. RNF40 was shown to be recruited to Egr2 target genes to specifically regulate myelination-related gene expression programs [51]. Together, these data suggest that H2B ubiquitylation plays integral roles in the proper development, regulation, and differentiation of neuronal precursors into glial cell subtypes as well as maintain overall nervous system health.

Given the importance of H2B ubiquitylation, it follows that deubiquitylation of histone H2B should offer complementary importance to RNF20/40 ubiquitylation. However, little is known about the roles of deubiquitylases —especially the ATXN7L3-dependent DUB complex components —in nervous system development, as loss of USP22, ENY2, or ATXN7L3 is embryonic lethal in mouse models [52–54] making in vivo studies difficult. Alternatively, we instead summarize the current knowledge on the role these ATXN7L3-dependent DUBs play in neural differentiation and development in vitro.

### USP22 and USP27X may influence proper neuronal differentiation

The role of USP22 and USP27X in mouse brain development was addressed by Kobayashi and colleagues [55]. Within the ventricular zone (VZ) and sub-ventricular zone (SVZ) of the developing mammalian brain are populations of neural stem cells and intermediate progenitors respectively that contribute to the development of the cerebral cortex. Kobayashi and colleagues used in utero electroporation of embryonic brains to assess neural progenitor migration patterns under USP22 or USP27X loss of function or overexpression conditions respectively. They found that loss of USP22 led to increases in neural progenitors in the intermediate zone and cortical plate and decreases in the presence of these cells in the VZ and SVZ after two days post electroporation. USP27X overexpression, however, led to a decrease in the number of neural progenitors in the intermediate zone and cortical plate while their numbers in the VZ and SVZ remained unchanged. These data suggest that while USP22 presence promotes differentiation, migration, and repopulation of VZ and SVZ neural progenitors, specific levels of USP27X governed whether neural progenitors underwent proliferation, migration, or differentiation within the developing brain [55]. It is worth mentioning that this study did not formally demonstrate the fidelity of USP22 knockdown or USP27X overexpression in the brain tissue analyzed, determine the expression of endogenous USP22 or USP27X proteins in the developing brain, nor investigate which cell types express these proteins. Furthermore, this study also did not assess if the observed phenotypes were due to changes in H2Bub1 regulation by USP22 or USP27X complexes. Therefore, the presence of endogenous USP22 and USP27X proteins in the developing cortex, and the mechanisms behind these results are yet to be defined.

### USP22 regulates neurogenesis upon brain injury

Neurogenesis occurs in the SVZ and dentate gyrus (DG) of the hippocampus in the adult mouse brain. The role of USP22 in this process upon brain injury was investigated by Zhang and colleagues. Using a traumatic brain injury (TBI) model in which direct force was applied to the exposed mouse brain, this group found that USP22 levels decreased one day post-TBI but increased three and five days post-TBI. Furthermore, USP22 is required for neurogenic and migration potential as demonstrated in neurosphere formation assays of neural stem cells obtained from mice post-TBI under USP22 loss or gain of function conditions. Consistently, USP22 knockdown mice show impaired performance in the Morris water maze test performed after TBI, indicating a requirement of USP22 for proper cognitive function upon brain injury. Together, these data reinforce USP22’s role in neurogenesis and cognitive function [56]. Yet again, these studies did not assess if regulation of H2Bub1 by USP22 or other mechanisms explained the phenotypes observed.

### USP22 regulates embryonic stem cell differentiation via H2Bub1 deubiquitylation at the *SOX2* promoter

The core factors governing embryonic stem cells (ESCs)’ pluripotent state include but are not limited to SOX2, OCT4, and NANOG [57, 58]. Sussman and team showed that USP22 repressed *Sox2* transcription via H2Bub1 deubiquitylation at the *Sox2* locus. During ESC differentiation via retinoic acid, USP22 levels increased while SOX2 levels decreased. This inverse correlation was observed not only for mouse ESCs, but for human as well – suggestive of a critical regulatory mechanism conserved across species. USP22 knockdown resulted in an increase in Sox2 mRNA while USP22 overexpression resulted in Sox2 downregulation. In fact, the relationship between USP22 protein levels and *Sox2* mRNA levels is deliberate, as the mRNA levels of other critical factors NANOG and OCT4 were not significantly downregulated by USP22 – with NANOG only being increased upon SOX2 reduction – nor was the H2Bub1 pattern altered within the *Oct4* gene locus [59]. Together, these data suggest the critical conserved role that DUBs such as USP22 and their orthologs play for epigenetic regulation of cell phenotypes. While these experiments indicate that USP22 regulates Sox2 transcription during pluripotency exit, given the heterogeneity of the differentiation protocols used, we cannot conclude that this mechanism occurs in neural cells. Whether the aforementioned dynamic between USP22 and *Sox2* persists during neuronal differentiation would be clarified by confirming these findings using neural stem cell to neuron differentiation or directed differentiation models such as iPSC-derived NGN2-induced neurons. Thus, the role of USP22 activity on gene regulation via H2Bub1 deubiquitylation remains understudied from a neurodevelopmental context and represents a potential avenue of research to uncover novel molecular mechanisms impacting neuronal differentiation lineages.

## ATXN7L3-dependent DUB complex components are mutated in neurodevelopmental disorders

Consistent with the importance of H2Bub1 in transcriptional regulation, recent studies have linked genetic disruption of ATXN7L3-dependent DUB complex components to neurodevelopmental disorders. Below, we summarize the main findings, implications and limitations of recent reports of gene variants in components of ATXN7L3-dependent DUB complexes that are associated with neurodevelopmental disorders.

### Disruptive *USP27X* variants are associated with X-linked intellectual disorder 105

**Fig. 2:**
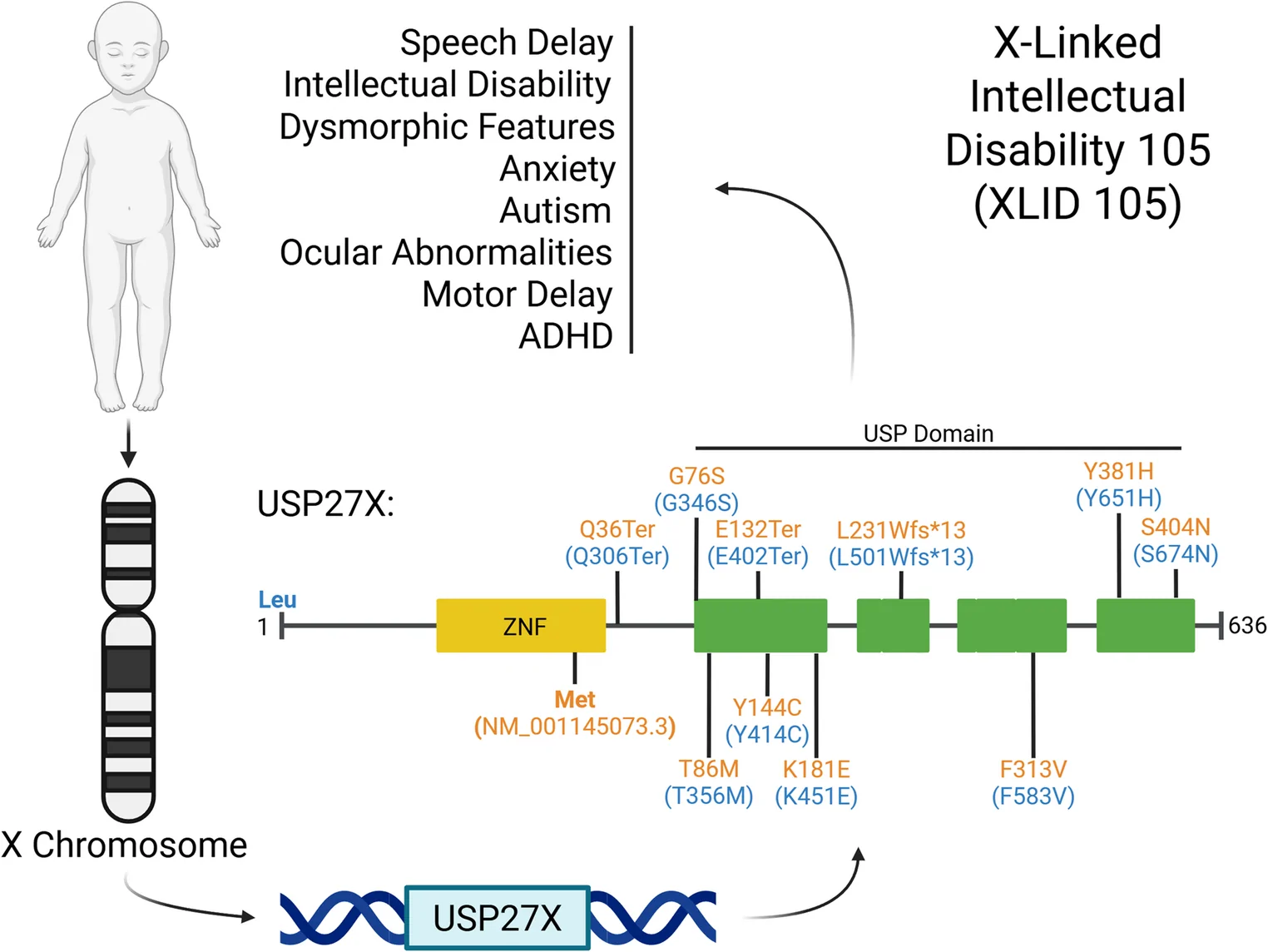
X-linked intellectual disorder 105 is associated with pathogenic variants in the *USP27X *gene. Individuals with XLID 105 present with the indicated symptoms that appear in early childhood and last for life, with symptom management as the only treatment option. These individuals harbor variants in the *USP27X* gene, located in the X-chromosome. These variants lead to missense or nonsense variants. *USP27X* missense variants result in amino acid changes – predominantly within the USP domain (green) – which yield a catalytically defective protein. Gene variant numbering in orange denote those documented with the canonical ATG start codon (consistent with the reference sequence NM_001145073.3), whereas the same variants in blue parentheticals denote documentation from the physiological CTG start codon. Created in BioRender. A, A. (2026) https://BioRender.com/061pjyt [77]

X-linked intellectual disorder 105 (XLID 105) was annotated in the Mendelian Inheritance of Man database from a report of two families with patients presenting with intellectual disability and other symptoms (OMIM #300984). Within an unbiased study – aimed to the discovery of several new XLID-associated genes, it was found that these patients carried variants in the *USP27X* gene [12]. The phenotypic spectrum and potential underlying mechanisms of XLID 105 were expanded by Koch and colleagues. They described nine additional families with eight novel inherited gene variants in the *USP27X* gene. Primarily affecting biological males (with sex chromosomes XY), these patients presented with a wide range of symptoms such as speech delay, intellectual disability, ADHD-related behaviors, dysmorphic features, autistic behaviors, motor delay, ocular abnormalities, or anxiety (Fig. 2). The variants p.Gln36Ter and p.Glu132Ter were predicted to produce USP27X loss of function, and the missense variants p.Gly76Ser, p.Tyr144Cys, p.Lys181Glu, p.Phe313Val, p.Ser404Asn, and p.Tyr381His were of particular interest as these patients presented similar phenotypes as the ones carrying loss of function variants [47].

Through ESC transfections and cycloheximide chase assays, it was found that the missense variants of *USP27X* did not alter the localization pattern or stability of the USP27X protein. Instead, catalytic activity was greatly reduced in variants p.Gly76Ser, p.Tyr144Cys, p.Phe313Val, p.Tyr381His, and p.Ser404Asn as indicated through K48 and K63 diubiquitin cleavage assays. This reduction in catalytic efficacy was inferred to be the basis of USP27X’s pathogenicity in XLID 105. Further mechanistic details of these defects were obtained using DUB activity-based probes, which label the nucleophilic attack involved in DUB catalysis [60, 61]. However, variants in a region of the protein that was solvent-exposed and not buried within the USP domain structure, such as p.Ser404Asn revealed something unexpected. This variant was still able to disrupt USP27X catalytic activity, leading to the hypothesis that allosteric recognition as well as direct ubiquitin binding by the USP domain contributes to the full DUB capability of USP27 × [47]. In the case of p.Ser404Asn, it was postulated that it works as an alpha helix cap that when mutated, could alter the folding of this local secondary structure. However, these studies were performed with the short isoform of USP27X, which negates the near-entirety of the N-terminus of the protein – a potential confounder when assessing the extent of variant pathogenic severity. Furthermore, these assays were performed with human USP27X transiently transfected in mouse ESCs, potentially obfuscating novel disease phenotypes had the cell model and protein source been of the same species, or a neurodevelopmentally-relevant model.

Just recently, a novel missense *USP27X* variant was characterized in a three-year-old male p.Thr86Met via trio-whole-exome sequencing, with a phenotype consistent with XLID 105 – notably intellectual disability, speech and motor delay, autistic behaviors, and craniofacial abnormalities (Fig. 2). This rare variant resides in the conserved catalytic domain of USP27X juxtapose to the catalytic cysteine at position 87. ChimeraX analysis of the mutation yielded alternate hydrogen bonding patterns that the Luo research team postulated may yield a destabilized catalytic core. These results were substantiated with functional assays demonstrating easier protein degradation of the p.Thr86Met compared to wild-type (WT) [62]. Another novel characterization made recently involves a case study of a six-year-old boy, where exome sequencing revealed a potential pathologic frameshift *USP27X* variant p.Leu231Trpfs*13. However, further characterization of this variant remains to be documented [63] (Fig. 2).

As mentioned previously, ATXN7L3 and ENY2 are the key modulators of true DUB activity of the USP enzymes [41]. A co-immunoprecipitation study found that the missense variants p.Gly76Ser and p.Tyr381His led to decreases in USP27X-ATXN7L3 interaction, while p.Tyr144Cys led to an increase. Therefore, it was hypothesized that drastic loss or gain of ATXN7L3 interaction, in addition to compromised catalytic activity of USP27X, could play a role in the pathogenicity of *USP27X* variants in XLID 105 [47]. Ultimately, a larger sample size, and the use of cellular or animal models to understand the impact of XLID 105 variants in endogenous H2Bub1 levels and in neurodevelopment will be needed to fully understand XLID 105 pathogenesis.

### A novel maternally inherited variant of USP51 is associated with a neurodevelopmental disorder

In 2024, a novel *USP51* variant was characterized by Garg et al. in a 19-year old biological male (with sex chromosomes XY) with autism spectrum disorder (ASD). Via trio-whole exome sequencing, this maternally inherited variant was termed p.Glu32GlyfsTer25, resulting in a frameshift-mediated truncation of USP51. Symptoms include intellectual disability, epilepsy, and absent speech [64]. Identification of additional individuals harboring *USP51* variants with overlapping phenotypes, as well as functional studies investigating the impact of these variants on USP51 activity and downstream cellular functions are warranted to confidently associate USP51 with the observed phenotypes.

### *De novo* variants In *ATXN7L3* are associated with a neurodevelopmental disorder

**Fig. 3:**
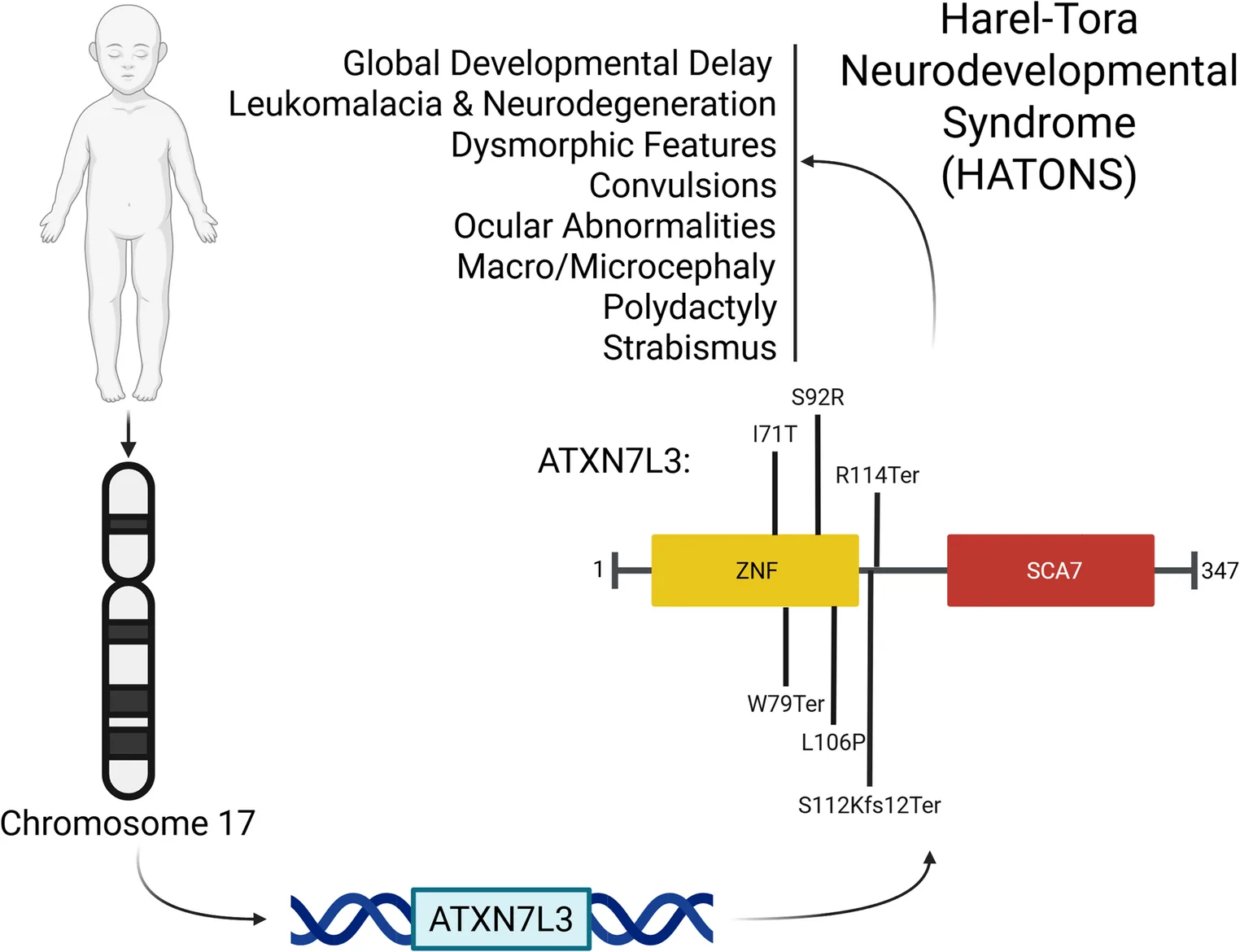
Harel-Tora neurodevelopmental disorder is associated with heterozygous *de novo *variants in the *ATXN7L3* gene. Individuals with Harel-Tora neurodevelopmental disorder present with the indicated symptoms and harbor heterozygous de novo variants in the *ATXN7L3* gene, located in chromosome 17. Truncation variants are predicted to lead to complete loss of ATXN7L3 protein function due to loss of the SCA7 domain and subsequent aberrant protein folding, while missense pathological variants yield an aberrant ZNF domain which may impair DNA binding and interactions with ENY2 or USPs for proper DUB activity. (ATXN7L3 map adapted from [65]). Created in BioRender. A, A. (2026) https://BioRender.com/vy6kawz [78]

A clinical study identified individuals with neurodevelopmental disorders from nine independent families, harboring heterozygous *de novo* variants in the *ATXN7L3* gene [48]. This condition was named Harel-Tora neurodevelopmental syndrome by OMIM (#621377). Of the five *ATXN7L3* variants identified, two of them, p.Arg114Ter and p.Ser112Lysfs12Ter, are predicted to generate truncated versions of the protein and hence result in loss-of-function. Symptoms of the individuals with these variants included global developmental delay, variable cerebral malformations such as leukomalacia or demyelination, craniofacial abnormalities, and hypotonia (Fig. 3). In these truncating variants, the C-terminal half of the protein is lost, including the spinocerebellar ataxia 7 (SCA7) domain which is important for chromatin interaction. Using patient-derived fibroblasts harboring the *ATXN7L3* p.Arg114Ter variant, it was demonstrated that the predicted truncated protein is not detectable, and mRNA is degraded by nonsense mediated decay, but these cells express about half of ATXN7L3 protein compared with control, consistent with heterozygosity of this variant. *ATXN7L3*-variant cells also display elevated total H2Bub1 levels. In case that the p.Ser112Lysfs12Ter truncated protein was expressed, it was postulated that due to the proximity of this C-terminal half of the protein to the zing-finger motif (Znf-Sgf11) which modulates overall SAGA DUB activity, perturbations caused by this *de novo* variant may yield aberrant folding affecting the ZNF domain and hence, compromised DUB activity [48].

Furthermore, two missense variants *ATXN7L3* p.Ser92Arg and p.Leu106Pro lead to single amino acid substitutions within the ZNF domain of the protein while a third missense variant p.Ile71Thr leads to a single amino acid change in the region between the ZNF and ENY2 binding domains of ATXN7L3. Since these variants are within conserved regions of the protein, it is suggested that these missense variants will affect protein function. Symptoms of individuals with these variants are similar to those mentioned previously in addition to presentation of possible convulsions and more severe cerebral malformations such as vermis atrophy or aberrant brain matter proportions [48]. Recently, a novel *de novo* variant p.Trp79* was identified in a 6.5-year-old boy, leading to premature truncation of the protein. Symptoms included developmental delay, strabismus, polydactyly, hypotonia, dysmorphic features, and abnormal brain MRI [66] (Fig. 3). These findings suggest a role of ATXN7L3 during human development requiring further investigation. Consistent with the phenotypes of humans with Harel-Tora neurodevelopmental syndrome, homozygous deletion of *Atxn7l3* in mice resulted in neurologic defects by embryonic day 8.5, with death shortly after. Heterozygous mice, however, were phenotypically normal [52, 67]. Ultimately, direct analysis of the impact of *ATXN7L3* missense variants on USP interaction and activation, and global H2Bub1 levels, together with the development of cellular models carrying these variants will be required to fully elucidate the role of ATXN7L3 in neurodevelopment and which steps of this process are affected upon genetic disruption in humans.

## Future directions

Here we summarized the literature associated with the role of H2Bub1 DUB complex components in neurodevelopment and human disease. This knowledge is very limited, and a large body of research is still needed to understand how these regulators participate in the development of the human nervous system – especially regarding the direct link between ATXN7L3, USP27X and USP51 neurodevelopmental disorder variants and H2Bub1 regulation, which is still unclear. These efforts will require identification of larger cohorts of patients carrying variants in *USP22*, *USP27X*, *USP51*, *ATXN7L3*, *ENY2* and others but there remains the question of whether any of these genes are essential for life. According to gnomAD v4.1.2, ATXN7L3 and USP22 have a pLi score of 1, ENY2 has a pLI of 0.91, and USP51 has a pLi score of 0.88, while USP27X has a gnomAD v2.1.1 pLi score of 0.92, indicating that these genes are highly constrained and intolerant to loss-of-function genetic variation in humans. This suggests that full gene dosage is important for human physiology. This may explain why variants in ENY2 or USP22 have not been found in patients with neurodevelopmental disorders. However, a more direct investigation of their role in human development will be required to explain this observation.

The current knowledge of the roles of ATXN7L3-dependent DUB complex components in neural development is still weak and is based on preliminary observations. A systematic analysis of the roles and downstream mechanisms orchestrated by these genes either H2Bub1-dependent or independent will be required for a confident association with neurodevelopment. For this, detailed study of their expression patterns, identification of the relevant cell types involved in their functions, generation of cellular or animal models of their depletion and systematic research of their role in brain development will be required.

It is unclear if H2Bub1 is regulated by ATXN7L3-dependent DUB complexes in human neural cells. Given the exquisite tissue specificity of DUB-substrate partnerships, this confirmation is required to fully define the role of H2Bub1 deubiquitylation in human neural development. This will be enabled by human induced pluripotent stem cell (iPSC) culture and differentiation with gene editing techniques. Engineering of knockout iPSCs lacking ATXN7L3-dependent DUB components or knockin iPSCs carrying gene variants found in humans will be key models to study their functional impact in the differentiation and function of individual neural cell types or in tissue-like differentiation models such as organoids or assembloids. The identification of the genes and transcriptional programs regulated by each component will require chromatin and transcriptional profiling techniques such as H2Bub1 chromatin immunoprecipitation (ChIP)-SEQ or CUT&Tag, combined with RNA-SEQ. Together, these approached will enable identifying the developmental programs and cellular processes regulated by H2Bub1 to better understand how their disruption drives neurodevelopmental disorders.

There remains the possibility that USP22, USP27X and USP51 target substrates beyond histone H2B in neural cells. Identification of these additional neural-specific substrates will be enabled by state-of-the-art techniques such as quantitative proteomics, genetic screens with CRISPR/Cas9 and in vitro ubiquitylation assays. The same applies to known USP22 substrates such as MYC, Androgen receptor, HES1, Xeroderma Pigmentosum group C protein or USP27X substrates such as cGAS, CBX2, Cyclin D1, cFLIPL, Cyclin E, SNAIL, BIM, RIG-I, HES1 and SETD3 that have been discovered in non-neural cell types [49, 52, 55, 68–75].

Key open questions are how do *USP27X*, *USP51* and *ATXN7L3* variants affect H2Bub1 levels in neural cells, if this leads to alterations in downstream gene expression and hence, yields neurodevelopmental disorder manifestation. Future studies aiming to answer these questions will aid in defining therapeutic targets and development of disease-modifying therapies such as enzyme replacement or small-molecule intervention. In summary, the existence of developmental disorders driven by disruption of ATXN7L3-dependent DUB complex components strongly suggests a role of H2Bub1 modification and its regulation in human neural development. However, extensive research is needed to definitively establish this connection.

## Data Availability

No datasets were generated or analysed during the current study.
